# Evaluation of data discretization methods to derive platform independent isoform expression signatures for multi-class tumor subtyping

**DOI:** 10.1186/1471-2164-16-S11-S3

**Published:** 2015-11-10

**Authors:** Segun Jung, Yingtao Bi, Ramana V Davuluri

**Affiliations:** 1 Division of Health and Biomedical Informatics, Department of Preventive Medicine, Northwestern University Feinberg School of Medicine, Chicago, IL 60611, USA

**Keywords:** RNA-seq, exon-array, platform transition, data discretization, multi-class classification

## Abstract

**Background:**

Many supervised learning algorithms have been applied in deriving gene signatures for patient stratification from gene expression data. However, transferring the multi-gene signatures from one analytical platform to another without loss of classification accuracy is a major challenge. Here, we compared three unsupervised data discretization methods--Equal-width binning, Equal-frequency binning, and *k*-means clustering--in accurately classifying the four known subtypes of glioblastoma multiforme (GBM) when the classification algorithms were trained on the isoform-level gene expression profiles from exon-array platform and tested on the corresponding profiles from RNA-seq data.

**Results:**

We applied an integrated machine learning framework that involves three sequential steps; feature selection, data discretization, and classification. For models trained and tested on exon-array data, the addition of data discretization step led to robust and accurate predictive models with fewer number of variables in the final models. For models trained on exon-array data and tested on RNA-seq data, the addition of data discretization step dramatically improved the classification accuracies with Equal-frequency binning showing the highest improvement with more than 90% accuracies for all the models with features chosen by Random Forest based feature selection. Overall, SVM classifier coupled with Equal-frequency binning achieved the best accuracy (> 95%). Without data discretization, however, only 73.6% accuracy was achieved at most.

**Conclusions:**

The classification algorithms, trained and tested on data from the same platform, yielded similar accuracies in predicting the four GBM subgroups. However, when dealing with cross-platform data, from exon-array to RNA-seq, the classifiers yielded stable models with highest classification accuracies on data transformed by Equal frequency binning. The approach presented here is generally applicable to other cancer types for classification and identification of molecular subgroups by integrating data across different gene expression platforms.

## Background

Molecular understanding of tumor heterogeneity is key to personalized medicine and effective cancer treatments. Numerous studies have identified molecularly distinct cancer subtypes among individual patients of the same histopathological type by performing a high-throughput gene expression analysis of the patient tumor samples [1]. While microarray-based gene expression estimates are often not precise or quantitative enough for applications in the clinical setting, the expression profile data from microarrays are the basis for the widely used OncotypeDX (Genomic Health, Redwood City, CA) test, which predicts the risk of recurrence in patients with early stage breast cancer [2]. The OncotypeDX assay analyzes the expression of 21 genes by RT-qPCR to provide a recurrence score that is unique to each patient [3,4]. More recently, the development of next-generation sequencing (NGS) based techniques, RNA-Seq [5], is enabling gene expression analysis to yield a much greater resolution for accurate identification of different isoforms. While several genome-wide expression profiling studies have dramatically improved our collective understanding of cancer biology and led to numerous clinical advancements [6,7], the use of genomics based molecular diagnostics, such as OncotypeDX [8-12], in clinical practice still remains largely unmet for majority of human cancers [13].

A crucial step in the translation of gene signatures derived from high-throughput platforms is validation in a clinical setting, using robust and quantitative assay platforms (e.g., RT-qPCR based assay) without loss of any classification accuracy [14]. A major bottleneck in translating the prognostic or molecular subtyping statistical models is lack of adaptability of the derived models from one analytical platform to another. In other words, assuming that we have gene expression data for a set of tumor samples (with known subtype/class labels) from two different analytical platforms, "can a statistical model derived on data from one platform (e.g., microarray/exon-array) accurately predict the class labels using data from another platform (e.g., RNA-Seq) for the same patient samples?" While several normalization strategies, such as locally weighted scatter plot smoothing (*loess) *[15,16], rank and quantile normalization methods [17-19], have been successfully applied to eliminate systematic errors in data from a same platform, these methods are not appropriate for normalization of data from different profiling platforms (microarray, RNA-Seq and RT-qPCR) because of the differences in the data scales and magnitude. In such cases, researchers usually accept the normalized data in the original analyses, and harvest the list of differentially expressed (significantly up or down) genes from each study by rank ordering. Then, the genes are prioritized by comparing the lists of up- and down-regulated (or rank ordered) genes between studies, rather than comparing individual expression values. However, these pre-processing methods are not useful in developing platform-independent statistical models.

Data discretization is a popular data pre-processing step used in supervised learning for creating the training sets. Data discretization transforms continuous values of feature variables to discrete ones [21,22]. It can significantly impact the performance of classification algorithms in the analysis of high-dimensional data [23]. Different data discretization methods have been developed that can be categorized as: (1) supervised vs. unsupervised methods depending on the availability of class labels; (2) global vs. local methods considering all or only one feature to discretize; and (3) static vs. dynamic methods based on interdependency between attributes. Many discretization techniques have been applied to analyze gene expression data, for example, to devise a new approach to explore gene regulatory networks [24], and as a pre-processing step to improve classification accuracy using microarray data [25]. While the previous studies have used the discretization method as a pre-processing step to design and apply the statistical models on data from one platform (e.g., microarray), our goal in this study is to evaluate three unsupervised data discretization methods--equal width (Equal-W) binning, equal frequency (Equal-F) binning, and k-means clustering--in combination with different feature selection and machine learning methods for deriving the most accurate classification model from one platform (e.g., exon-array), and apply it to data from another platform (e.g., RNA-seq) for molecular subtype prediction of a future cancer patient.

Feature selection algorithms seek for a subset of relevant features to use in model construction in order to simplify and reduce over-fitting of the models. The wrapper, filter, and embedded methods are the three main categories that have been widely used in biomedical research to deal with a large feature space [26,27]. Briefly, wrapper algorithm uses a predictive model that scores on a new each subset to train, and test on the remaining set; filter algorithm uses a direct measure instead of the error rate estimate to score a feature subset; embedded algorithm integrates feature selection as part of the model construction process including the Recursive Feature Elimination (RFE) algorithm. In this study, we adopted two advanced feature selection algorithms based on SVM and RF, and one filter method using the coefficient of variation (CV), a statistical measure to find highly variable genes.

Using a subset of most important genes (variables/features) screened by the variable selection methods, numerous classification methods have been applied to tackle disease sample classification problems. For example, SVM was applied for characterizing functional roles of genes in yeast genome and cancer tissues [28,29], RF for classifying cancer patients and predicting drug response for cancer cell lines [30-32], NB (naïve Bayes) for classification on prostate cancer [33,34], and PAM (Prediction Analysis of Microarrays) for molecular classification of brain tumor and heart disease [35,36]. These studies, however, focused largely on the data from one platform such as microarray, although cross-platform data analysis would help find robust gene signatures. Recently, we developed PIGExClass [20], platform-independent isoform-level gene expression based classification system, that captures and transfers gene signatures from one analytical platform to another through data discretization. PIGExClass is an integrative system that consists of data discretization, feature selection, and classification. The application of PIGExClass has led to the development of a novel molecular classifier (or gene panel) for diagnosis of GBM subtypes [20]. Motivated by the importance of data discretization step in PIGExClass algorithm, in this paper we evaluated the performance of three data discretization methods together with four popular machine learning algorithms to derive reliable platform-independent multi-class classification models; specifically, predicting the four known subtypes of GBM patient samples from the same platform as well as independent platforms.

## Results

### Data-discretization retained the classification accuracy with fewer number of variables for data from same platform

Because gene isoforms (variables) whose expression levels do not vary much across the samples are less useful for discriminating the four GBM subtypes, We selected 2,000 isoforms with the highest variability across the samples, using CV (coefficient of variation). To search for an optimal bin number *k *for the discretization, we explored various bin sizes including the optimal bin number (*k *= 11) based on Dougherty's formula [37], and chose the bin number of *k *= 10 as it consistently achieved good accuracy. Then we applied two advanced feature selection algorithms, SVM-recursive feature elimination (SVM-RFE) [38] and RF based feature selection (RF_based_FS) [39], and prepared independent training and testing datasets by dividing the exon-array samples into four fold; 3/4^th ^(257 samples) for training and 1/4^th ^(85 samples) for testing. We describe below the classification performance for each variable selection method--CV, SVM-RFE and RF_based_FS.

**Figure 1:**
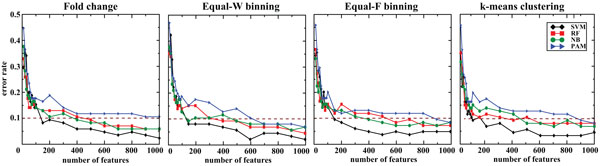
**Accuracy of classifiers on the same platform with features ranked by the CV**. Independent exon-array data of 257 and 85 samples are used for training and testing, respectively. The dotted brown line marks 90% accuracy.

First, we trained the classifiers with the features ranked by the CV that represent high generic variability. Overall, the accuracy of the derived classifiers was within the range of 89.4-97.6% for FC and 91.3-97.6% for discretized data (Figure 1 and Table 1), suggesting that the discretization retained the classification accuracy of the respective models. More importantly, SVM achieved similar accuracy with Equal-W binning using only 500 features in comparison to without discretization. For RF, NB and PAM the classification accuracies and the number of variables used in the models did not differ significantly between the discretized and non-discretized data. We then trained the classifiers by considering only the top 100 features that can be clinically testable by, for example, RT-PCR. We observed that SVM with *k*-means clustering yielded the best accuracy of 90.6% (Table 2).

**Table 1:** Comparison of classification accuracy on the same platform using top ranked gene isoforms.

Feature selection	CV (#)				SVM-RFE (#)				RF_based_FS (#)			
**Classifier**	**FC**	**Equal-W**	**Equal-F**	***k*-means**	**FC**	**Equal-W**	**Equal-F**	***k*-means**	**FC**	**Equal-W**	**Equal-F**	***k*-means**

**SVM**	97.6 (1000)	97.6 (600)	96.4 (500)	96.4 (600)	97.6 (1000)	95.2 (600)	97.6 (1000)	97.6 (1000)	**98.8 (150)**	96.4 (70)	96.4 (200)	**98.8 (150)**

**RF**	94.1 (900)	95.2 (1000)	92.9 (800)	91.7 (600)	**96.4 (900)**	96.4 (1000)	95.2 (400)	96.4 (1000)	95.2 (300)	94.1 (150)	92.9 (500)	92.9 (400)

**NB**	94.1 (700)	94.1 (900)	92.9 (600)	92.9 (700)	83.5 (1000)	83.5 (1000)	88.2 (80)	83.5 (1000)	95.2 (300)	95.2 (200)	95.2 (600)	**96.4 (400)**

**PAM**	89.4 (900)	92.9 (1000)	91.7 (1000)	91.7 (1000)	87.0 (900)	83.5 (900)	87.0 (900)	87.0 (900)	92.9 (600)	**94.1 (200)**	94.1 (800)	92.9 (400)

# Number of variables in the classification model

Comparison of classification methods both trained (257 samples) and tested (85 samples) on exon-array data. The best accuracy (percentage of samples correctly predicted) achieved by each combination of the four classifiers and three feature selection schemes are presented, with number of features used in the best fitted model is shown in parenthesis. The models were built by stepwise addition of feature variables into the model by considering the top 1,000 ranked feature variables. Best accuracy, achieved with the least number of features, is marked in bold for each classification method.

**Table 2:** Comparison of classification accuracy using top 100 genes using data from same platform.

Feature selection	CV				SVM-RFE				RF_based_FS			
**Classifier**	**FC**	**Equal-W**	**Equal-F**	***k*-means**	**FC**	**Equal-W**	**Equal-F**	***k*-means**	**FC**	**Equal-W**	**Equal-F**	***k*-means**

**SVM**	84.7	85.9	85.9	90.6	77.6	81.2	92.9	87.1	**96.5**	94.1	95.3	**96.5**

**RF**	85.9	85.9	84.7	85.9	81.2	78.8	88.2	87.1	91.7	**92.9**	91.7	90.6

**NB**	82.3	81.2	80.0	80.0	75.3	69.4	81.2	78.8	90.6	**92.9**	85.9	84.7

**PAM**	85.9	87.1	85.9	84.7	71.7	70.6	84.7	80.0	**91.7**	**91.7**	87.1	85.9

The classification models were trained (257 samples) and tested (85 samples) on exon-array data. Highest accuracy for each classification method is marked in bold. While SVM in combination with RF_based_FS performed best whit the highest accuracy for both FC data (without discretization) and k-means discretised data, the other three classifiers (RF, NB and PAM) in combination with RF_based_FS achieved comparable classification accuracies on Eaual-W discretized data.

**Figure 2:**
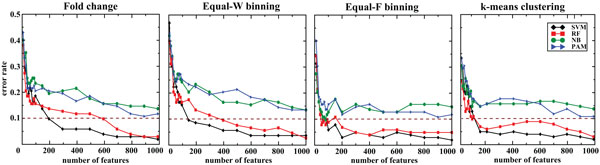
**Classification performance on the same platform using features ranked by SVM-RFE**. We evaluated the classification algorithms using exon-array data for both training (257 samples) and testing (85 samples). The dotted line indicates 90% accuracy.

Second, we evaluated the classification performance using the features ranked by SVM-RFE. Accuracy of the classifiers ranged from 83.5 to 97.6% for both FC and discretized data (Figure 2 and Table 1). Again, SVM showed similar accuracy between discretized and FC data, but required lot fewer variables in the model that was trained on Equal-W binning data. Similarly, RF showed similar accuracy between discretized and non-discretized data, but the RF model trained on Equal-F binning data used only 400 variables in comparison to 1,000 variables required for FC data. Interestingly, NB not only improved the classification accuracy with Equal-F binning data but also used much fewer number of variables (80 in comparison to 1,000) to achieve the higher accuracy. For PAM, the classification accuracy and number of variables in the models remained similar between FC and discretized data. Using the top 100 features, SVM still attained the best accuracy with Equal-F binning (Table 2).

**Figure 3:**
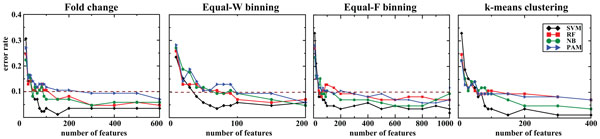
**Prediction accuracy of the classifiers on the same platform using features selected by RF_based_FS**. We evaluated the four classifiers using exon-array data for both training (257 samples) and testing (85 samples). The dotted line denotes 90% accuracy.

Lastly, we used the features selected by RF_based_FS to assess the classifiers' performance. Accuracy of the classifiers did not fluctuate much by staying within the range of 92.9-98.8% for both non-discretized and discretized data (Figure 3 and Table 1). Overall, all the classifiers tested retained their highest accuracies, but with significantly fewer number of variables in the final models. While SVM achieved the best accuracy (98.8%) with FC, it retained comparable accuracy (96.4%) with just 70 variables in the model trained on Equal-W binning data in comparison to 150 variables in the model trained on FC data. Similarly, both RF and NB models trained on Equal-W binning data achieved similar accuracy with fewer number of variables in comparison to FC data. Interestingly, PAM model trained on Equal-W binned data slightly improved the accuracy with lot fewer variables in comparison to FC data.

In summary, all the classifiers trained and tested on the discretized data from same platform resulted with lot fewer number of variables, yet retaining the high accuracies in comparison to the corresponding models that were trained on FC data. Overall, while SVM achieved the best accuracy, Equal-W discretization in combination with RF_based_FS helped build the classification models with significantly lower number of variables in the final models.

### Data discretization improved cross-platform predictions

In order to evaluate the accuracy of classification models on data derived by different gene-expression platforms (exon-array and RNA-seq in this study), we trained the classifiers using the data from exon-array and tested on matched RNA-seq datasets for the same TCGA samples. First, we observed that the classification framework resulted in poor classification accuracies when the classification and feature selection algorithms were trained on FC data from exon-array data and tested on corresponding FC data from RNA-seq platform (Table 3). The best accuracy of 73.6% on FC data was achieved by RF with RF_based_FS with just 40 variables in the final model. However, with data discretization we observed significant improvements in the performance of the classification framework. Below, we report the classification performance in more detail based on testing of the models on data from 76 RNA-seq samples.

**Table 3:** Comparison of classification accuracy using top ranked features for platform transition

Feature selection	CV (#)				SVM-RFE (#)				RF_based_FS (#)			
**Classifier**	**FC**	**Equal-W**	**Equal-F**	***k*-means**	**FC**	**Equal-W**	**Equal-F**	***k*-means**	**FC**	**Equal-W**	**Equal-F**	***k*-means**

**SVM**	43.4 (500)	35.5 (80)	**100 (700)**	92.1 (300)	51.3 (400)	75.0 (200)	100 (1000)	73.6 (60)	48.6 (20)	39.4 (50)	97.3 (600)	92.1 (200)

**RF**	69.7 (300)	84.2 (1000)	97.3 (1000)	89.4 (600)	61.8 (60)	89.4 (700)	96.0 (1000)	81.5 (100)	73.6 (40)	85.5 (100)	**97.3 (800)**	88.1 (300)

**NB**	27.6 (800)	30.2 (10)	92.1 (500)	75 (200)	35.5 (40)	38.1 (10)	85.5 (600)	67.1 (60)	35.5 (200)	34.2 (20)	**94.7 (600)**	78.9 (90)

**PAM**	44.7 (300)	26.3 (10)	92.1 (400)	76.3 (300)	44.7 (900)	39.4 (600)	89.4 (400)	60.5 (60)	46.0 (10)	34.2 (10)	**93.4 (500)**	82.8 (200)

# Number of variables in the classification model

Comparison of classification methods trained on exon-array (342 samples) and tested on RNA-seq (76 samples). The best accuracy (percentage of samples correctly predicted) achieved by each combination of the four classifiers and three feature selection schemes are presented, with number of features used in the best fitted model is shown in parenthesis. The models were built by stepwise addition of feature variables into the model by considering the top 1,000 ranked feature variables. Highest accuracy, achieved with the least number of features, for each classification method is marked in bold.

**Figure 4:**
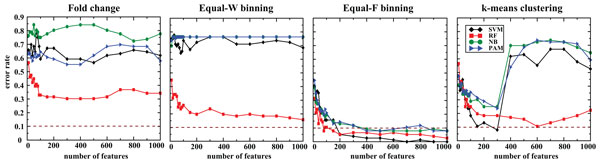
**Accuracy of classifiers for independent platform with features ranked by the CV**. 342 samples of exon-array and 76 samples of RNA-seq were used for each training and testing to predict the four GBM subtypes. The dotted brown line marks 90% accuracy.

For CV based feature selection, the classification accuracy of the models trained on FC (without discretization) was rather poor and ranged from 27.6 to 69.7% (Figure 4 and Table 3). However, the accuracy of the models built on Equal-F binning data achieved higher and stable accuracy, ranging from 92.1 to 100%. Notably, the SVM classifier accomplished the best accuracy of 100% (700 features) with Equal-F binning followed by *k*-means with SVM (92.1% accuracy; 300 features). Equal-W binning improved the accuracy for RF (84.2% accuracy; 1000 features), but not for the other three classifiers. When using the top 100 features in the final model, RF with Equal-F binning correctly predicted 68 samples out of 76, achieving ~90% accuracy (Table 4).

**Table 4:** Comparison of classification accuracy using top 100 features for platform transition.

Feature selection	CV (%)				SVM-RFE (%)				RF_based_FS (%)			
**Classifier**	**FC**	**Equal-W**	**Equal-F**	***k*-means**	**FC**	**Equal-W**	**Equal-F**	***k*-means**	**FC**	**Equal-W**	**Equal-F**	***k*-means**

**SVM**	40.8	26.3	**84.2**	81.6	36.8	40.8	85.5	39.5	28.9	30.2	76.3	39.5

**RF**	67.1	73.7	89.5	76.3	55.2	60.5	86.8	80.2	56.6	81.6	**90.8 **	85.5

**NB**	25.0	23.7	80.2	71.0	32.9	23.7	76.3	22.3	23.7	23.7	**84.2**	36.8

**PAM**	35.5	23.7	78.9	64.5	39.5	27.6	73.7	32.9	39.5	23.7	**81.6**	44.7

The classification models were trained on exon-array (342 samples) and tested on RNA-seq (76 samples) data. Highest accuracy for each classification method is marked in bold. Only RF with Equal-F binning achieved greater than 90% classification accuracy.

**Figure 5:**
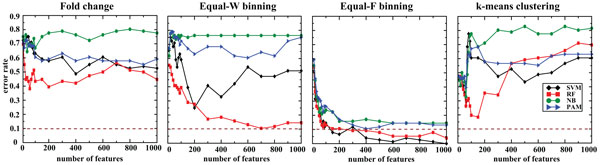
**Classification accuracy for independent platform with features chosen by SVM-RFE**. 342 samples of exon-array and 76 samples of RNA-seq were used for each training and testing to predict the four GBM subtypes. The dotted line indiciates 90% accuracy.

Similarly, for SVM-RFE features selection, the prediction accuracy of the models on FC data is quite low, within the range of 35.5-61.8%. While Equal-F binning improved the accuracy of all the four classifiers, Equal-W binning improved the accuracy for SVM and RF only (Figure 5 and Table 3). Most notably, with Equal-F binning discretization, SVM classifier achieved the highest accuracy using 1,000 features. For both Equal-W binning and *k*-means clustering discretization, RF achieved the best performance. Using the top 100 features, RF with Equal-F binning achieved 86.8% accuracy that is 31.6% higher than the best accuracy with FC (55.2%).

**Figure 6:**
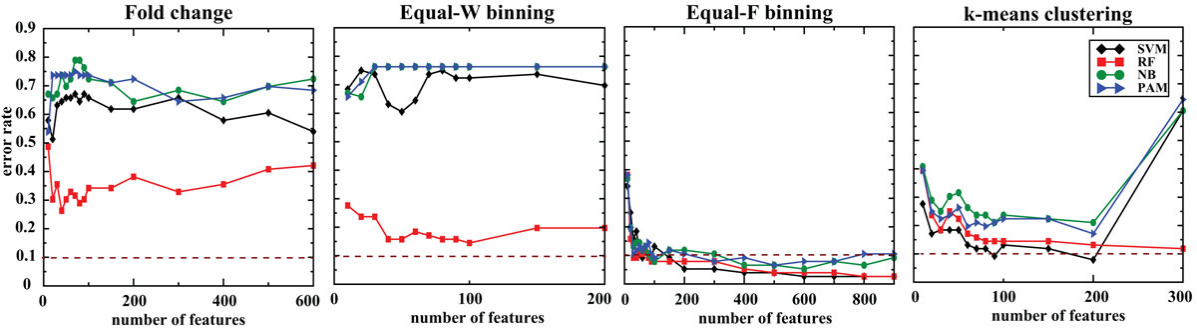
**Accuracy of classifiers for independent platform with features selected by RF_based_FS**. 342 samples of exon-array and 76 samples of RNA-seq were used for training and testing to predict the four GBM subtypes. The dotted line denotes 90% accuracy.

For RF_based_FS, the classification accuracies were dramatically improved for the models trained on discretized data, with Equal-F binning showing the highest improvement with more than 90% accuracies for all the models (Figure 6 and Table 3). Models built using k-means based discretized data also showed significant improvement with fewer number of variables in the final models. Considering only the top 100 features, RF with Equal-F performed 90.8% (69/76 samples) accuracy whereas RF with FC correctly predicted only 43 samples out of 76 (Table 4). We present the sensitivity and specificity measures for each classifier trained on the top ranking 100 features from exon-array data and tested on corresponding data from RNA-seq in Table 5.

**Table 5:** GBM subtype prediction.

Class	PN		N		CL		M	
**Method**	**Sn**	**Sp**	**Sn**	**Sp**	**Sn**	**Sp**	**Sn**	**Sp**

**SVM**	0.681	0.963	0.818	1.000	0.772	0.926	0.863	0.870

**RF**	0.833	0.965	0.944	1.000	0.888	0.965	0.833	0.982

**NB**	0.950	0.946	1.000	0.910	0.850	0.928	0.800	0.910

**PAM**	0.937	0.916	0.875	0.966	0.875	0.966	0.750	0.983

Sensitivity (Sn) and Specificity (Sp) of the classifiers trained on the top 100 feature variables from exon-array data and tested on the independent RNA-seq data for prediction of the four GBM Subtypes.

In summary, we found that Equal-F binning based discretization performed best, followed by *k*-means clustering based data discretization. Equal-W binning improved only for RF and not for other classifiers for cross-platform class label predictions.

## Discussion

The evaluation of the three unsupervised discretization methods using our integrated classification framework revealed that the addition of discretization step into the learning framework led to a large average increase in classification accuracy for all the classification models trained on data from one gene expression platform and tested on corresponding data from a different platform. Specifically, the best method, Equal-F binning, improves performance of all the classifiers and feature selection methods for cross-platform transfer of the derived models.

In machine learning, data discretization is primarily used as a data pre-processing step for various reasons, for example, (1) for classification methods that can handle only discrete variables, (2) for improving the human interpretation, (3) for faster computation process with a reduced level of data complexity, (4) for handling non-linear relations in the data, e.g., very highly and very lowly expressed genes are more relevant to cancer subtype, and (5) to harmonize the heterogeneous data. In this study, we showed that simple unsupervised discretization indeed improved the classification accuracy by harmonizing the data that come in different scale and magnitude from different gene expression platforms. The discretization step lead to numerically comparable measures of gene expression between different platforms, and translate the classification models (consisting of multiple transcript variables) across platforms. However, the discretization methods applied in this study have some limitations. For example, Equal-W binning is prone to outliers that may skew the distribution [37]. The *k*-means discretization performed relatively well with the CV and RF based feature selection schemes. The known drawback of this clustering discretization, however, is in choosing initial cluster centroids which in general is randomly assigned and less robust to outliers; additionally, it is sensitive to the number of clusters affecting classification accuracy. The Equal-F binning performed superior in this study and appeared to be feasible.

The choice of classification algorithms is often important dealing with a certain dataset as each of the algorithms has its own strengths and weaknesses. We experimented on the four state-of-the-art machine learning approaches based on maximum margin, decision tree, probabilistic and clustering classification. While SVM achieved the best accuracy, the performance of RF was more consistent when tested with various numbers of features and data types. We used the linear kernel SVM because it is known to be less prone to overfitting than nonlinear kernels such as radial basis function (RBF); intuitively, the RBF kernel could perform better when the data is linearly not separable or the feature and sample spaces are well balanced. PAM and NB also performed fairly well with the features chosen by RF_based_FS. NB is known to be robust with irrelevant features, but the performance would be quickly degraded when correlated features are added.

## Conclusions

**Figure 7:**
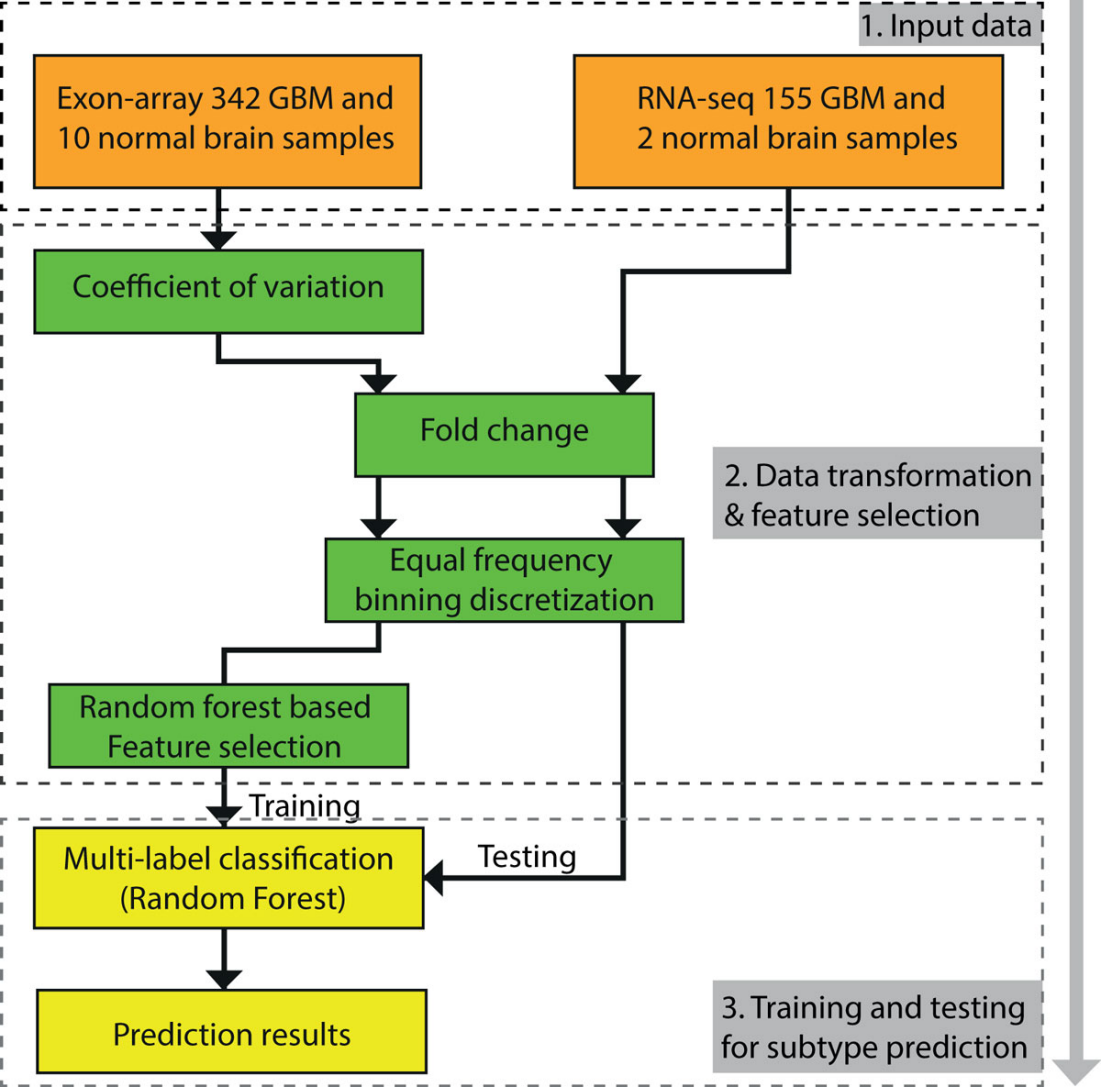
**Proposed computational pipeline**. Based on our experiment, we proposed a computational procedure to build a molecular classifier for GBM patient subtyping prediction.

For training and testing the models on data from same platform, all the classifiers built with features selected by RF_based_FS led to robust and accurate predictive models regardless of the data format. While data discretization step does not significantly improve the accuracy of the classifiers, it significantly reduced the number of variables in the final models. For cross-platform training and testing of the classifiers, Equal-F binning outperformed FC, Equal-W binning and *k*-means clustering. With Equal-F binning, RF_based_FS identified important features more efficiently than the CV and SVM-RFE when fewer gene isoforms are considered in classification. Based on these encouraging results, we propose an integrative machine learning framework that involves feature selection, data discretization, and classification model build up by training and testing for independent platform (Figure 7). We anticipate that the application of this machine-learning framework, which includes data discretization as a key step, will provide quantitative and reproducible stratification of cancer patients with prognostic significance, the potential to improve precision therapy and the selection of drugs with subtype-specific efficacy. More importantly, the approach presented here is generally applicable to other cancer types for classification and identification of molecular subgroups.

## Methods

### Dataset

We obtained isoform-level gene expression estimates and molecular subtype information for 342 and 155 GBM samples profiled by Affymetrix exon-arrays and RNA-seq, respectively. The four molecular subgroups are neural (N), proneural (PN), mesenchymal (M) and classical (CL). Gene expression profiles for 76 (18 are N; 22 are PN; 16 are M; and 20 are CL subtype) samples were available from both RNA-seq and exon-array platforms. The common samples were used to assess classification performance for platform transition. We followed the data pre-processing procedure and obtained patients' GBM subtype information (class labels) from our recent study [14]; briefly, we downloaded the unprocessed Affymetrix exon-array dataset of 426 GBM samples and 10 normal brain samples from TCGA data portal (https://tcga-data.nci.nih.gov/tcga/); obtained the isoform expression of 114,930 transcript variants (equivalent to 35,612 genes) using the Multi-Mapping Bayesian Gene eXpression program [33]. The estimated expression values were then normalized across the samples, using the locally weighted scatterplot smoothing algorithm [34], a non-parametric regression method. To select 2,000 most variable transcripts, we applied Pearson's correlation coefficient with cutoff of > 0.8 followed by the CV. See [14] for more details.

### Data type and transformation

We processed the gene expression data to estimate the FC values, and then three unsupervised discretization techniques--Equal-W binning, Equal-F binning, and *k*-means clustering--on the continuous FC data.

FC is a measure of a quantitative change of gene expression, defined by FC=log_2 _(*T*/*N*), where *T *is estimated expression values of a tumor sample and *N *is median expression of normal brain samples.

To determine the number of bins for discretization, Dougherty *et al *[28] suggested a heuristic to set the maximum number of bins *k *= max (1, 2 log (*l*)), where *l *is the number of distinct values of the attribute. Boulle [29] proposed an algorithm to find an optimal bin number for Equal-F and Equal-W, and demonstrated the optimal bin number performs similar to the bin number *k *= 10, considered as a default for most cases. While the former approach resulted the maximum bin number *k *= 11, we extensively evaluated by exploring various bin numbers of *k *= 2*i *for *i=*{1, 2, ..., 10}.

Equal-W binning algorithm seeks for maximum and minimum values, and then divides the range into the user-defined equal width intervals defined as Equal-W=(max(GE(*i*))-min(GE(*i*)))/number of bins, where GE is isoform-level transcript gene expression of sample *i*. Then, continuous variables are assigned into the corresponding bin numbers.

Equal-F binning algorithm sorts all continuous variables in ascending order, and then divides the range into the user-defined intervals so that every interval contains the same number of sorted values defined as Equal-F=sort(GE(*i*))/number of bins.

*K*-means clustering algorithm calculates distance-based similarity to cluster the continuous variables. With the user-defined number of clusters, the algorithm iteratively finds centroids until no data point is reassigned to the updated centroids.

### Feature selection methods

To capture most significant features, we first applied Pearson's correlation to the normalized expression data with a cutoff value of 0.8. Second, we used the CV to assess the degree of variability for each transcript.

CV is defined as CV=*σ */*µ*, where *σ *and *µ *are the standard deviation and mean, respectively. Based on the CV scores, we selected the top 2000 transcripts out of ~115,000. To refine the selected features further, we employed two advanced feature selection algorithms based on SVM and RF that iteratively evaluate each feature's contribution to the classification performance. We adopted the programs available in R packages 'mSVM-RFE' and 'varSelRF.'

SVM-RFE is a feature search algorithm that measures feature's importance to the data by iteratively eliminating one feature at a time [13]. Adopted from the weight vector **w **of the binary classification problem, the ranking criteria is the coefficients of $w_{i}^{2}\left(i=1,...,n\right)$; features with the highest weights are the most informative. Thus, the procedure of SVM-RFE is composed of training the SVM classifier, computing the ranking criteria ${\text{w}}_{i}^{2}$ for all features, and eliminating the feature with the lowest ranking criterion. This process is repeated until a small subset of features is achieved.

RF_based_FS method uses both backward elimination strategy and the importance spectrum to search a set of important variables [31]. Concisely, multiple random forests were iteratively constructed to search for a set of variable in each forest that yields the smallest out-of-bag (OOB) error rate. The main advantage of this method is that it returns a very small set of genes while retaining high accuracy.

### Classification methods

We considered the four classification methods--SVM, RF, NB, and PAM--to compare the performance on platform transition using the 76 GBM samples.

SVM is primarily a two-class classifier that constructs a hyperplane to separate the data with maximum margin [35,36]. For multiclass classification problems, two techniques are widely used: one is to build one-versus-all classifiers, and choose the class that yields maximum margin for test examples; the other is to build a set of one-versus-one classifiers. For class *C *> 2, *C *(*C*−1)/2 binary classifiers are trained and the appropriate class is determined by major voting. In this study, we used the latter approach with a linear kernel method as the size of features is larger than samples.

RF is an ensemble learning method that builds decision trees with binary splits [37]. Each tree is grown randomly in two steps. First, a subset of predictors is chosen at random from all the predictors. Second, a bootstrap sample of the data is randomly drawn with replacement from the original sample. For each RF tree, an unused observation is utilized to calculate the classification accuracy.

NB is a simple probabilistic classification method grounded in Bayes' theorem, for calculating conditional probabilities, with an independence assumption [38]. For a given instance (example), the NB classifier calculates the probability belonging to a certain class. The basic underlying assumption is that the features (*x*_1_,*..*.,*x_n_*) of an instance *X *are conditionally independent given the class *C*. For example, for a class *C *that maximizes the likelihood is P(*X*|*C*)*=*P(*X_1_,...,X_n_*|*C*). The conditional independence enables the conditional probability as a product of simpler probabilities defined by P(*X*|*C*)*=*Π P(*X_i_*|*C*).

PAM is a sample classification method that uses the nearest shrunken centroid approach for transcript-variants gene expression data [26]. Briefly, the method computes a standardized centroid for each class. Then, it shrinks each of the class centroids by removing genes toward the overall centroid for all classes using a user-defined threshold. A new sample is assigned to the nearest centroid for which classification is based on the unseen sample's gene expression profile.

### Accuracy

We estimated the overall classification accuracy based on the number of correct predictions divided by the total number of prediction samples defined as ACC=(number of correct predictions)/(total number of test samples). In addition, sensitivity (Sn) and specificity (Sp) for each sub-group (one GBM sub-group vs the rest of the GBM groups together) are calculated as $\text{Sn}=\frac{\sum_{i=1}^{n}tp_{i}}{\sum_{i=1}^{n}tp_{i}+fn_{i}}$ and $\text{Sp}=\frac{\sum_{i=1}^{n}tn_{i}}{\sum_{i=1}^{n}tn_{i}+fp_{i}}$ where *tp_i_, tn_i_*, and *fn_i_*, are true positive, true negative, false positive, and false negative for class *C_i_*, respectively.

## Competing interests

The authors declare that they have no competing interests.

## Authors' contributions

SJ participated in the design of the project, performed the bioinformatics and statistical analysis, and drafted the manuscript. YB and RVD conceived the idea and participated in the design of the project. SJ, YB, and RVD revised the manuscript. All authors read and approved the final manuscript.

## Declarations

The publication costs for this article were funded by the NIH-NLM grant R01LM011297.

This article has been published as part of *BMC Genomics *Volume 16 Supplement 11, 2015: Selected articles from the Fourth IEEE International Conference on Computational Advances in Bio and medical Sciences (ICCABS 2014): Genomics. The full contents of the supplement are available online at http://www.biomedcentral.com/bmcgenomics/supplements/16/S11.
